# Cost-effectiveness of procalcitonin-guided antibiotic duration for hospitalized patients with sepsis

**DOI:** 10.1186/s13054-025-05732-w

**Published:** 2025-11-28

**Authors:** Matt Stevenson, Jessica E. Forsyth, Anower Hossain, Ranjit Lall, Paul Dark, ADAPT-Sepsis collaborators, Gordon Carlson, Michael J. Naisbitt, Dominic Kay, Sharon Barber, Jane Parez, Stephanie Lee, Reece Doonan, Lisa Swindells, Jessica Pendlebury, Jessica Holden, Jacob Hadfeild, Jay Naisbitt, Andrew Claxton, Emma Parkin, Daniel Horner, Anila Sukumarian, Benedict Gyamerah, Alice Duncombe, Chris Taylor, Wadhi Habeichi, Joanna Borzomato, Paul Ferris, Justin Roberts, Jonathan Clayton, John Macdonald, Redmond P. Tully, Andrew Drummond, Joy Dearden, Michelle Mulcahy, Carrole Blessen, Angiy Vian-Michael, Grainne O’Connor, Jonathan Scargill, Sarah Winnard, Jonathan C. Clayton, Ingeborg D. Welters, Karen Williams, David Shaw, Victoria Waugh, Julie Patrick-Heselton, Carol Paisley, Suzannah Phillips, Emily Johnson, Jaime Fernandez Roman, Maria Lopez Martinez, Arra Mahiya, Daniel Watkin, Zachary Thomas, Andrew Davison, Sofia Farina, Maria Norris, Silvia Manes, Jin-Xi Yuan, Josh Colfar, Caitlin Lythgoe, Ibrahim Almafreji, Lisa Bailey, Robert J. Parker, Ian Turner-Bone, Laura Wilding, Michaela Lloyd, Harriet Murrant, Leanne Smith, Ben Morton, Dylan Middleton, William McCaig, Amie Hughes, Lucy Fuller, Gavin Perkins, Jo Gresty, Ellie Reeves, Teresa Melody, Helen Taylor, Sara Bawa, Celinamma Maliaykal, William McIver, Mohamed Mooradun, Tayyibah Mohamed, Heather Willis, Aidan Butler, Amy Hooper, Adam Boulton, Lucie Linhartova, Rachel Smith, Daniel Park, Anna Dennis, Joyce Yeung, Hozefa Ebrahim, Neil Crooks, Sarah Milton-White, Sarah Ballinger, Natalie Carling, Julia Sampson, Chloe Norman, Marie Thomas, Alexandra Nance, Emma Evans, Lesley Horton, Tony Whitehouse, Amy Clark, Liz Ratcliffe, Anna Casey, Natalie Dooley, Karen Ellis, Christopher Sheridan, Lisa Moody, Puja Mulholland, Rachel Webster, Samantha Harkett, Sophia Beddows, Caroline Dooley, Eamonn Reda, Stephanie Goundry, Elaine Spruce, Tonny Veenith, Dhruv Parekh, Natalie Walmsley, Malikah Saba, Rebecca Gill, Esther Thomas, Jaimin Patel, Mansoor Bangash, Alistair Roy, Anthony Rostron, Lindsey Woods, Sarah Cornell, Kimberley Rogerson, Jo Boxall, Trina Burns, Susan Cooper, Kirstie Knight, Sarah Constable, Laura Kenny, Jonathan Vernazza, Maxwell Richardson, Matthew Bickerton, Andrew Camplejohn, Roseanne Cope, Julie Sheriff, Simon Whiteley, Max Ridley, Richard Gould, Sarah Perrin, Elizabeth Wilby, Susan Trott, Sheila Salada, Sarah Watts, Zoe Friar, Bethan Ogg, Clare Howcroft, Kate Long, Helena Baker, Jessica Watson, Jodie Sedgwick, Ronan McMullan, James McNamee, Danny McAuley, Peter McGuigan, Kathyrn Ward, Justine Ward, Aisling O’Neill, Stephanie Finn, Erin Collins, Jackie Green, Christine Turley, Gerard Quinn, Matthew Adams, Chris Wright, Gareth McKeeman, Rory Loughlin, Lynsey Reilly, Heather Gallagher, Steve Coward, Rachel Morrison, Tom Lawton, Amy Robinson, Rafaq Azad, Megan Kitching, Chris Cockcroft, Louise Akeroyd, Jackie Todd, Susan Kimachia, Lisa Burgess, Shereen Bano, Thomas P. Hellyer, Iain J. McCullagh, Stephen Hamilton, Arti Gulati, Benjamin Brown, Stephanie Grieveson, Tara Shrestha, Ian Storey, Fatima Simoes, Verity Calder, Carole Hays, Maite Babio-Galan, Deborah White, Jennifer Collins, Leigh Dunn, Susan Taylor, Luzgie Gavina, Tessa Wilkinson, Pamela Garcia, Lauren Butler, James Savage, Jayne Harwood, Sarah Cook, Jonathan Shelton, Ian Clement, Samantha Burn, Nathan Carroll, Lauren Clark, Lauren Ferguson, Sam Foggett, Alison Hague, Tracy Handley, Chelsea Hewison, Samantha Kitchener, Laura McEachan, Nicole McEwen, Ryan Perry, Olivia Purdy, Amie Thomson, Stephanie Williams, Madhu Balasubramaniam, Rebecca Flanagan, Emma McKenna, Scott Latham, Danielle Hollick, Glykeria Pakou, Helder Filipe, Margaret McNeil, Sara Mingo, Catarina Veiga, Poh Choo Teoh, Amitaa Maharajh, Mark De Neef, Coral Cole, Saida Solkar, Oghenemega Okotete, Mervyn Singer, Chris Wilson, David Brealey, Niall MacCallum, Ingrid Hass, Georgia Bercades, Deborah Smyth, Gladys Martir, Anna Reyes, Maria Alexandra Zapata Martinez, Laura Gallagher, Hodan Omer, Jonathan Bannard-Smith, Deborah Paripoorani, Charlotte Taylor, Gregory Yates, Katharine Wylie, Sadiya Salah, Fionn Quilty, Timothy W. Felton, Pete D. G. Alexander, Luke Ward, Joanne Rothwell, Sherly George, Hannah Currie, Katharine Sellers, Aditya Adhikarla, Jayanthi Rajasekar, Sarah Purvis, Isha Garg, Vijay Jagannathan, Hillie Corr, Magda Stefaniak, Simon Sinclair, Ceri Lynch, Bethan Gibson, Piroska Toth-Tarsoly, Zolton Auer, Daniel Law, Jayparaska Singh, Shaun Oram, Arsalan Zaki, Alan Dodd, Bethan Deacon, Lisa Roche, Leannne Jones, Justyna Smeaton, Kevin Agravante, Oladele Akinyoola, Keri Turner, Joanne Pugh, Sam Evans, Lisa Mellish, Angharad Rees, Geraint Lewis, John Burke, Nia Bromage, Nicola Llewellyn, Justine Evans, Marlo Constantino, Chinari P. K. Subudhi, Celia Chu, Katy Edwards, Rashmi Gupta, Carolyn Williams, Lindiwe Mashangombe, Misbah Javed, Alistair Morris, Julia Sampson, Alexandra Gibbins, Celia Peters, Megan Meredith, Alice Baddeley, Anthony Baidoo, Dan Harvey, Alexandra McCoy, Sophie Lubbock, David Ford, Amy Clark, Laura Lloyd, Mohammed Habib, Gemma Clark, Ian King, Carla Goode, Angela Sellick, Shova Sharma, Anita Assi, Jennifer Dengate, Matthew Grice, Laura Brammer, Chinyere Ajonu, Samuel Redgate, Wendy Smith, Manjinder Khakh, Hannah Howson-Wells, Simon Atkin, Max Casbon, Frances Olsen, Bhavin Mistry, Victoria Mitchinson, Aysha Talbot, Jonathan Clayton, Shondipon Laha, Sushruth Madanapalli Raghunath, Alex Jude Fonseca, Alexandra Williams, Sacha Connor, Rebecca Allock, Lisa Halsall, Sean McMullen, Jonathan C. Clayton, Fiona Jutsum, Laura Price, Susie Pajak, Hannah Gibson, Mark Bates, Karen Reid, Alison Bland, Alison Stephens, Denise Donaldson, Mandy Beekes, Jo Stickley, Colene Adams, Amy Bowes, Sanal Jose, Nikolaos Manetas-Stavrakakis, Rebecca Wilcox, Rachel Rikunenko, Maria Houghton, Andrew Donnelly, Julie Summers, Christopher Mowatt, Katherine Richmond, Rebecca Jones, Tammy Davie, Michael Barnsley, Mick Pursglove, James Pearce, Philippa Lane, Natasha Cole, Megan Phillips, Joanna Jasiewicz, Sarah Hamad, Jyoti Sharma, Muhammad Adeeb, Mohammad Qasim, Lisa Barnsley, Louise Walton, Claire Wright, Helen Kaura, Cathy McCormack, Dave Latham, Emily Wynne, Catherine McCormack, Kathy Richmond, Claire Gabriel, Richard Lowsby, Elijah Matovu, Katherine Pagett, Deborah Maren, Tenifayo Adeyemo, Hannah David, Mark Quiambao, Joanne Harold, Sheron Clarke, Valery Gurov, Anthony Mkandhla, Chris Duff, Helen Duce, Dumindu Karangoda, Estefania Treus, Leanne Howard, Georgina Luck, Alex Ross, Mark Belsham, Shatha Abu-Hijleh, Christopher Thompson, Minnie Gellamucho, Jane Delaney, Michelle Davies, Elisa Sernicola, Lucy Bailey, Angelene Cope, Claire Hudson, Umar Mohyddin, Jack Lee, Ben Storey-Waters, Faye Halliday, George Mathew, Sarah Birch, Craig Denmade, Sarah White, Rosie Clare, Ya-Wen J. Huang, Dawn Herbert, Lynn Rowbottom, Madeleine McGrady, Nazir I. Lone, Timothy S. Walsh, Maria Amamio, Gillian Andrew, Lucy Barclay, Sophie Birch, Kate Briton, Lisa-Marie Butt, Sarah Clark, Jessica Crossan, Katherine Doverman, Michael Gillies, David Griffith, David Hope, Kallirroi Kefala, Ian Laurenson, Nazir Lone, Lucy Macdonald, Craig Marshall, Corrienne McCulloch, Gregor McNeill, Nicola Rea, Scott Simpson, Jo Singleton, Joanne Thompson, Elzbieta Czarniak, Olga Lucia Moncayo Nieto, Stephen Bonner, Jeremy Henning, Amy Thompson, Jessica Jones, Steven Liggett, Craig Mower, Sarah Bircham, Bobette Kamangu, Abiola Shoremekun, Anitha Ratnakumar, Keith Hugill, Matt Thomas, Paul Thomas, Ruth Worner, Borislava Borislavova, Kati Hayes, Beverley Faulkner, Emma Gendall, Elizabeth Goff, Hayley Blackmore, Mike Spivey, Sarah Bean, Karen Burt, Anthea Patterson, Lisa Vipond, Matthew Martin, Nick Vallotton, Samuel Waterman, Michael Kelsey, Ana Zuriaga-Alvaro, Loreta Mostoles, Anezka Pratley, Nabeel Amiruddin, Helen Ashby, Gemma Wood, George Priestley, Phil Antil, Joseph Carter, David Yates, Rob Ferguson, Harriet Carter, Zoe Cinquina, Belinder K. Ghuman, Uzma Manazar, Gillian Richards, Hannah C. McNeil, Keith K. Young, Anthony C. Gordon, Paul R. Mouncey, Peter Wilson, Dewi Williams, Catherine Jardine, Karen Craik, Julia Anderson, Laura Wild, Stacey Cotterall, Mandy Austin, Mark Wilkinson, Niall Harbison

**Affiliations:** 1 Sheffield Centre for of Health and Related Research, University of Sheffield, 30 Regent Street, Sheffield, S1 4DA UK; 2 Warwick Medical School, Clinical Trials Unit, University of Warwick, Coventry, UK; 3 Division of Immunology, Immunity to Infection and Respiratory Medicine, University of Manchester, Critical Care Unit, Northern Care Alliance NHS Foundation Trust, Salford Care Organization, Stott Lane, Salford, UK

**Keywords:** Sepsis, Procalcitonin, Antibiotics, Antibiotic duration, Cost-effectiveness, Health economics

## Abstract

**Background:**

Procalcitonin (PCT)-guided antibiotic duration for critically ill adults with sepsis may be clinically effective and safe. However, cost-effectiveness analyses using clinical trial data for this precision medicine approach in critical care are lacking. This economic evaluation investigates the cost-effectiveness of a daily PCT-guided protocol to guide the duration of antibiotic treatment in adult patients with sepsis.

**Methods:**

Two analyses were conducted, the first estimating the cost per quality-adjusted life year (QALY) of the ADAPT-Sepsis study, which recruited 2760 patients randomized to a daily PCT-guided protocol, a daily C-reactive protein-guided protocol and standard care. The second analysis used meta-analyzed results from ADAPT-Sepsis and other PCT-guided treatment studies and employed a lifetime horizon. Key outcomes were the incremental costs and QALYs gained from using the daily PCT-guided protocol approach compared with standard care. Other outcome measures included changes in days of antibiotics, days of hospital stay, days of intensive care unit stay, the percentage of deaths and the number of PCT tests performed.

**Results:**

Cost-effectiveness results were driven by the assumed impact of PCT testing on mortality although the confidence/credible intervals for ADAPT-Sepsis and the meta-analyzed data both included no effect. Within ADAPT-Sepsis, the use of PCT tests cost €427 more per patient and was associated with a small QALY loss (0.001), which suggests the daily PCT-guided protocol is dominated. Using meta-analyzed data, the daily PCT-guided protocol was assumed to cost €330 more per patient but was associated with 0.139 more QALYs, resulting in a cost per QALY gained of €2384. If only antibiotic use and PCT tests were assumed to differ then PCT testing is estimated to cost no more than €110 per patient with QALYs equal in both arms regardless of whether ADAPT-Sepsis or meta-analyzed data were used.

**Conclusions:**

This economic analysis has shown that a PCT-guided protocol to guide the duration of antibiotic treatment could be cost-effective. Where only differences in antibiotic use and PCT testing are assumed, the increased costs per patient are modest which may be seen as worthwhile to safely improve antibiotic stewardship for critically ill adult patients with sepsis.

**Graphical abstract:**

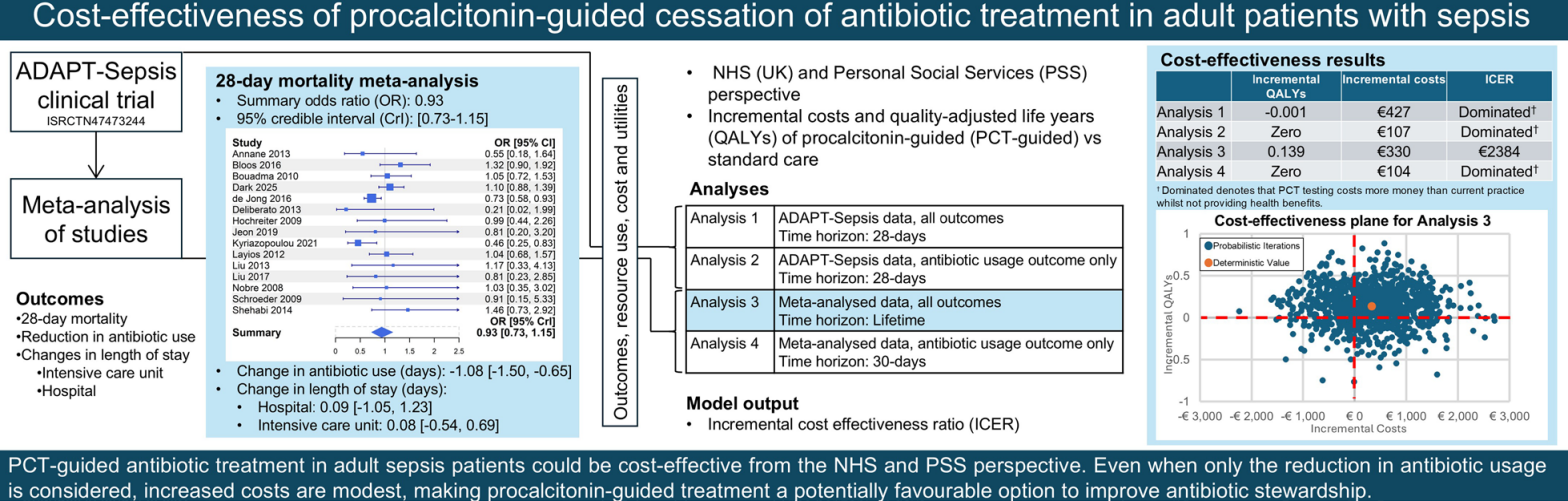

**Supplementary Information:**

The online version contains supplementary material available at 10.1186/s13054-025-05732-w.

## Background

Timely administration of appropriate antimicrobial therapy is a global standard in emergency sepsis care to ensure optimal patient outcomes. However, the ideal duration for antibiotic treatment for an individual patient with sepsis remains unclear, with discontinuation decisions predominantly guided by daily clinical assessments. Biomarker-guided discontinuation, especially using serial serum measurement of procalcitonin (PCT), has shown to safely reduce antibiotic use and may help improve patient outcomes. Despite this, clinical trial evidence has been judged to be low quality, leading to a weak international consensus recommendation for routine PCT-guided antibiotic discontinuation in sepsis care, and no consensus on other commonly monitored serum inflammatory markers such as C-reactive protein (CRP). Furthermore, there is an acknowledged lack of clinical trial data on the cost effectiveness of biomarker-guided antibiotic discontinuation protocols in sepsis care [1].

The ADAPT-Sepsis multi-center, three-arm, intervention-concealed individual patient randomized controlled trial (RCT) was UK government commissioned to determine the clinical and cost effectiveness of antibiotic discontinuation protocols based on daily serum PCT or CRP results compared with standard practice (the use of no tests). Multi-disciplinary antibiotic stewardship practice was a standard of care at each recruitment center for all critically ill patients with suspected sepsis [2, 3]. The ADAPT-Sepsis trial was adequately powered (sample size n = 2760) to detect clinically superior important differences in antibiotic duration when comparing biomarker protocols with standard care. The trial also contained sufficient precision to monitor patient safety based on a pre-specified non-inferiority margin. Notably, to improve study quality and contribute further to the available body of work that underpins the current international guidance, the ADAPT-Sepsis trial was uniquely designed to be intervention-concealed to minimize the risk of bias associated with this previous body of reported open-labelled studies.

The ADAPT-Sepsis trial completed recruitment in June 2024 and the clinical effectiveness results have been reported [4]. In summary, the daily PCT protocol significantly reduces the use of antibiotics for sepsis patients, while also meeting the non-inferior criterion for safety. The daily CRP protocol did not reduce the use of antibiotics for sepsis patients and was inconclusive for safety.

As a stated commissioning aim of the ADAPT-Sepsis trial, this paper aims to estimate the cost-effectiveness of biomarkers compared with standard care from a within study and an extrapolated lifetime horizon. Further analysis is undertaken meta-analyzing data from ADAPT-Sepsis with previously published randomized clinical trials reporting overall survival (OS), days of hospitalization, duration of stay in an intensive care unit (ICU) and duration of antibiotic treatment. Due to the lack of antibiotic reduction in the CRP arm and inconclusive evidence for its safety, only the daily PCT-guided protocol was compared with standard care in this cost-effectiveness analysis.

## Methods

### Overview of economic evaluations undertaken

A model was built to simulate the outcomes of patients admitted to hospital with sepsis. Key outcome measures were the proportion of patients alive at 30 days, the duration of antibiotic treatment and the length of hospital/ICU stay. A UK National Health Service (NHS) and Personal Social Services (PSS) perspective was adopted following National Institute for Health and Care Excellence (NICE) guidance [7]. These were pre-stated health-economic analyses as stated aims of the ADAPT-Sepsis trial’s National Institute for Health and Care Research funding commission [5, 6].

The cost-effectiveness of four analyses were evaluated. These were: Analysis 1, a within trial analysis using data from ADAPT-Sepsis at 28 days (the point of trial primary outcomes); Analysis 2, replicating Analysis 1, but assuming that the only differences between the daily PCT-guided protocol arm and standard care in ADAPT-Sepsis were in days of antibiotic use and days of PCT testing as these were the only statistically significant key outcomes; Analysis 3, meta-analyzing data from ADAPT-Sepsis [4] (at 28 days) with previously published studies of comparisons between PCT testing and the standard care/control arm (which typically report results at 28 or 30 days); and Analysis 4, replicating Analysis 3, but assuming that the only differences between the daily PCT-guided protocol arm and the standard care arm were in days of antibiotic use and days of PCT testing as these were the only statistically significant key outcomes. Analyses 3 and 4 used the duration of PCT costs from ADAPT-Sepsis [4] as insufficient data were reported in other studies. The study is reported following the Consolidated Health Economic Evaluation Reporting Standards (CHEERS) 2022 reporting guideline.

The cost-effectiveness outcome measure was modelled incremental cost-effectiveness ratio (ICER) which was provided as cost per quality-adjusted life year (QALY). The analysis was based on patient-level resource use and mortality observed in published literature providing estimates of costs and health-related quality of life. Both costs and QALYs were discounted at 3.5% per year [7]. The time horizon was 28 days for Analyses 1 and 2 as these were within-study analyses of ADAPT-Sepsis, was patient-lifetime for Analysis 3 as the QALY losses associated with differential mortality need to be fully considered, and was 30 days for Analysis 4, as it was assumed that PCT testing had no impact on mortality and data for the meta-analysis commonly reported outcomes at 30 days. Probabilistic results were generated in addition to deterministic results. All costs were calculated in UK pounds sterling (£) using 2023/2024 prices and converted to Euros (€) assuming that £1.00 = €1.161 [8]. However, the pre-stated analyses relate directly to the UK, and may not be generalizable to other jurisdictions, this is considered further in the discussion section.

None of the analyses included the societal gain from better antibiotic stewardship, as this is very difficult to quantify precisely and is an omitted benefit from the PCT arm in all analyses.

### Resource use and costs

For the PCT and control arms in the ADAPT-Sepsis trial we collected the average number of days in ICU, the average number of days in hospital, and the average duration of antibiotic use. For the PCT arm, the average number of PCT tests were collected.

The cost of a day in ICU was estimated from the National Schedule of NHS costs 2023/24 [9], assuming that the weighted average of XC01Z, XC02Z, XC03Z, XC04Z, XC05Z and XC06Z (Adult Critical care, one or more organs supported) was applicable resulting in a cost of £2302 (€2673). The cost for a general ward day was assumed to be the weighted average cost of DZ11R – DZ11V (lobar, atypical or viral pneumonia, without interventions) for non-elective short stay patients which was £673 (€781). The costs of PCT testing have been taken from a recent publication, Webb et al. [10], assuming that this value (£15.20 (€17.65)) included the costs required to interpret, or act upon, the test.

The costs of antibiotics use were estimated from the interventions used in ADAPT-Sepsis [4], from the British National Formulary [11] and from the drugs and pharmaceutical electronic market information tool (eMIT) [12]. Glucose solute, was also costed which was estimated to be £1.95 (€2.26) per the average defined daily dose (DDD), as reported by the World Health Organization, based on the average cost of 500 ml reported by eMIT [12]. The assumed cost per antibiotic day was lower-bounded by assuming that vial-sharing was allowed, which is not clinical practice, which resulted in a cost per antibiotic day of £14.79 (€17.17). The cost assuming no vial sharing was £16.37 (€19.01), however, this would likely be an over-estimate as each use was associated with the number of vials for the average DDD rather than accounting for variation. Considering these limitations, we decided to use a value of £16.00 (€18.58) per antibiotic day, assuming that this round number also included the costs of consumables associated with infusions. This cost was assumed applicable to both the daily PCT-guided protocol and standard care arms as the observed usage of antibiotics was similar between the arms. The drugs used in the first 28 days, together with the frequency of use, DDD when used, and the cost of a DDD are shown in supplementary material Appendix Table 1. In total, 27,939 antibiotic days were provided in the ADAPT-Sepsis RCT [4]. For Analysis 3, no additional long-term costs associated with the sepsis episode have been considered.

**Table 1:** Key clinical parameters for the health economic analyses

Changes associated with PCT testing	Mean estimate^a^ (95% credible interval)	
	Analyses 1 and 2^b^	Analyses 3 and 4^b^
Antibiotic use (days)	−0.88 (−1.58 to −0.19)	−1.08 (−1.60 to −0.65)
Hospital length of stay (days)	0.10 (−0.81 to 1.01)	0.09 (−1.05 to 1.23)
Intensive care unit length of stay (days)	0.11 (−0.69 to 0.91)	0.08 (−0.54 to 0.69)
Percentage of deaths (absolute difference)	1.57 (−2.18 to 5.32)	−1.10 (−4.56 to 2.39)
Number of PCT tests performed (units)	7.00 (6.60 to 7.40)	7.00 (6.60 to 7.40)

^a^ Parentheses are 95% confidence intervals for Analyses 1 and 2 and 95% credible intervals for Analyses 3 and 4

^b^ Analyses 2 and 4 use only parameters with a statistically significant difference: days of antibiotic use and number of PCT tests

### Utilities and QALYs

Utility is a measure of a patient’s quality of life, commonly used in cost-effectiveness models, with a value of 1 representing perfect health, and a value of 0 representing a state equivalent to death. QALYs are a combination of the utility multiplied by the time spent with this utility, with 1 QALY representing one year at perfect health. The age- and sex-matched cohort observed in ADAPT-Sepsis would have a utility (using the NICE recommended EQ-5D UK tariff) of 0.84 [13], which was assumed to apply when a patient was discharged. Being on a general ward was associated with a utility decrement of 0.58 taken from Hollman et al., [14] resulting in an estimated utility for patients in a general ward of 0.26. It was assumed that being in an ICU would be associated with a utility of zero. It was further assumed that patients who died within 28 or 30 days accrued no QALYs during the hospital stay.

Survivors at 30-days were assumed to accrue 12.68 discounted QALYs, which was estimated assuming general UK population life expectancy and utility for patients who survive the sepsis episode, with an average age of 60 years, and 40% of patients being female [15] (as observed in ADAPT-Sepsis [4]). Sensitivity analyses were conducted assuming a lower value (of 10 discounted QALYs) in case patients who survived were left with comorbidities.

### Summary of study data from ADAPT-sepsis trial

Data from ADAPT-Sepsis [4] relating to the following outcomes were used in the model: total antibiotic treatment duration to 28 days; all-cause mortality at 28 days; length of ICU stay; time to hospital discharge; and the number of days of PCT testing. Compared with control, the use of PCT significantly reduced mean antibiotic treatment duration by 0.88 days (95% confidence interval (CI) 0.19 to 1.58 days), non-significantly increased all-cause mortality by 1.57 percentage points (95% CI −2.18 to 5.32), non-significantly increased mean time spent in ICU by 0.11 days (95% CI −0.69 to 0.91) and non-significantly increased mean length of stay by 0.10 days (95% CI −0.81 to 1.01). On average, each person in the PCT arm required 7 PCT tests (standard error of the mean 0.20) compared with zero for the control arm (standard practice).

### Summary of study data from other relevant clinical trials

Data from ADAPT-Sepsis [4] were combined with previous studies of PCT-guided treatment compared with standard care/control within meta-analyses. Identification of previously-published relevant studies was made through the use of a recently published systematic review of PCT-guided interventions, Kubo et al. [16] and in a patient-level meta-analysis of randomized trials, by Wirz et al. [1] Wirz et al. [1] identified 11 studies [17–27], which were supplemented by six studies [28–33], in Kubo et al. [16].

The studies were assessed and three were excluded. These were: Oliveira et al. [19] as this reported a comparison of PCT vs CRP; Vishalashi et al. [32] as the study arms were imbalanced in relation to a key prognostic factor, septic shock, which was 82% in the control arm and 56% in the PCT-guided arm; and Xu et al. which reported a statistically significant difference in (nominal) *p*-value in Acute Physiology and Chronic Health Evaluation II score, a key prognostic factor, between the groups.

For OS, data that was closest to the 30-day mark were extracted from the individual papers and meta-analyzed using a random effects model (total number of participants (n) = 6216). For antibiotic use and length of stay (both hospital and ICU), insufficient reporting within the individual papers meant that, for each outcome, the meta-analyzed patient level data from Wirz et al. [1] (n = 4482) were combined with data from ADAPT-Sepsis [4] (n = 1842) totaling 6324 patients, using a fixed effects model, due to the small number of data sets. The difference in numbers of participants included in the analyses of OS versus other outcomes are due to the lack of outcome data from four studies [28–31], and different reported participant numbers in two studies [4, 26]. The results of the meta-analysis are shown in supplementary material Appendix Figs. 1 and 2. For Analysis 3, the odds ratios from the meta-analysis were applied to the proportion of deaths at 30 days using pooled data from the standard care arms within the meta-analysis, which was 20.9% (651/3115).

**Fig. 1:**
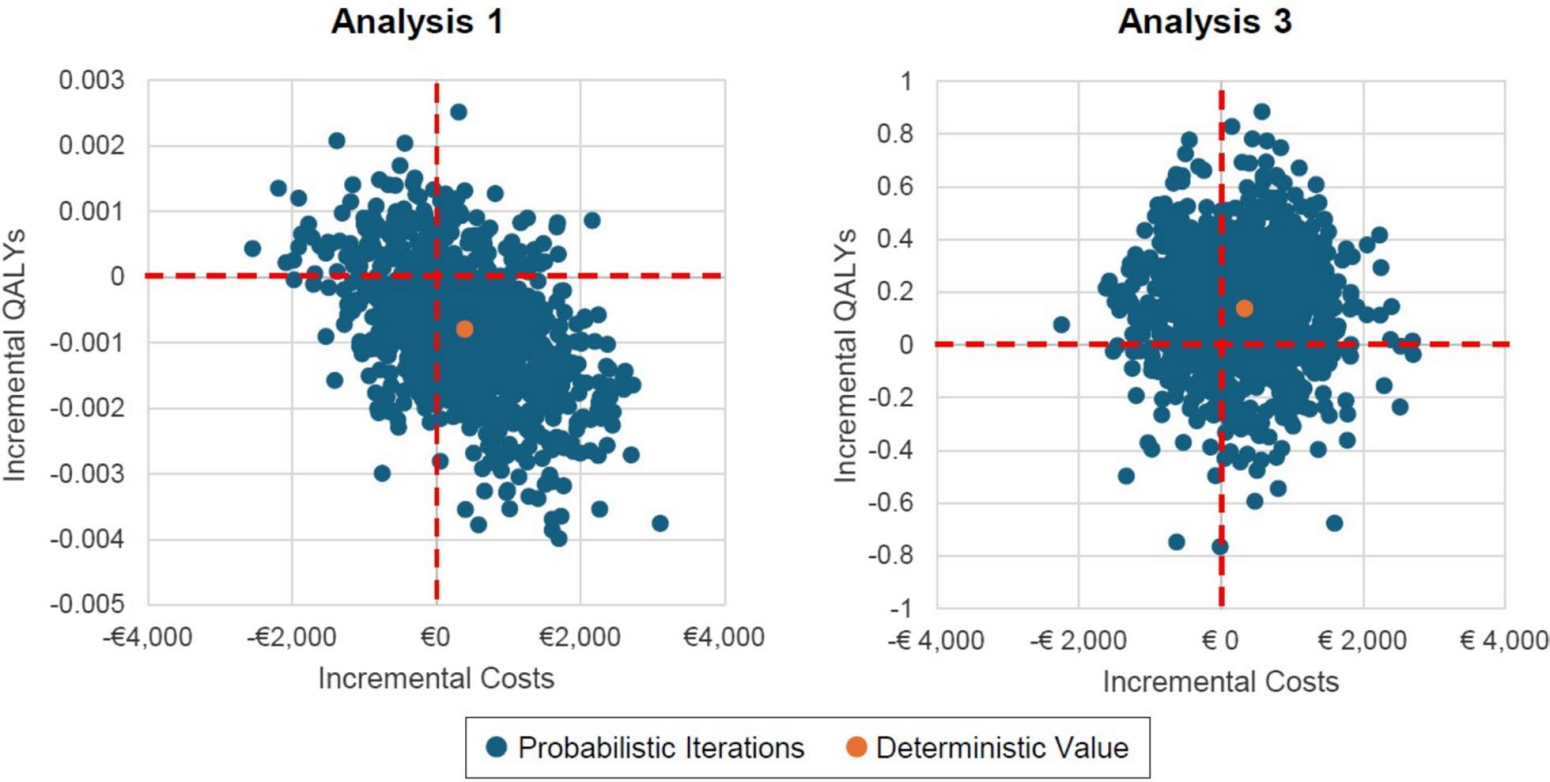
The cost-effectiveness planes for Analysis 1 and Analysis 3

**Fig. 2:**
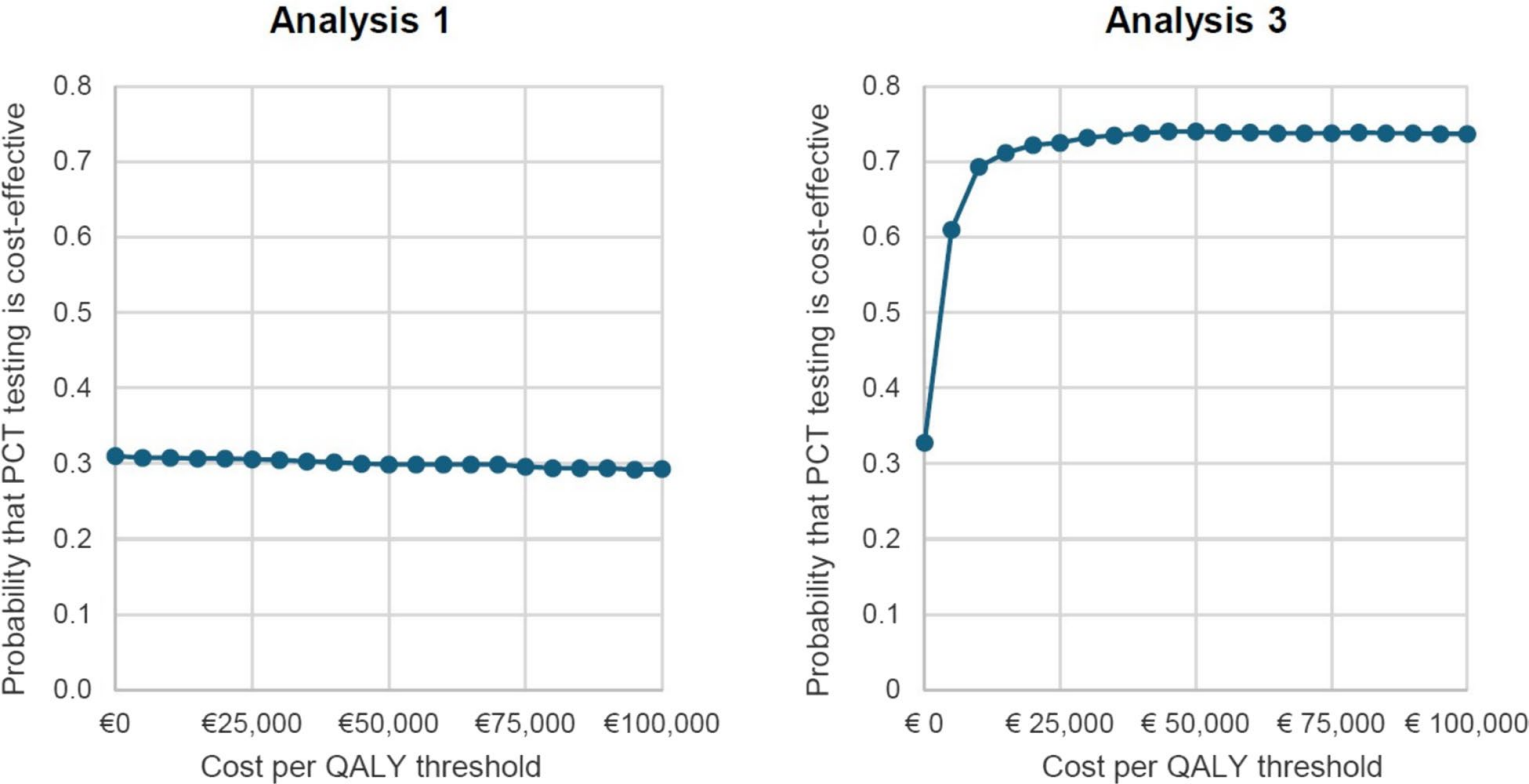
The cost-effectiveness acceptability curves for Analysis 1 and Analysis 3

### Summary of key clinical parameters used in the analyses

Table 1 provides the estimated changes in antibiotic use, hospital length of stay, ICU length of stay, percentage of deaths and number of PCT tests due to the adoption of PCT testing which are used within the health economic analyses.

### Generating cost-effectiveness results

Probabilistic results are presented for each analysis, as these were similar to the deterministic results and provide more detail on the uncertainty in the results. The following results are reported: the incremental costs of PCT testing; the incremental QALYs associated with PCT testing; the incremental cost-effectiveness ratio (ICER) in terms of cost per QALY gained; the probability that PCT testing produces more QALYs; and the probability that PCT testing is cost-effective at a £20,000 (€23,220) per QALY gained threshold.

## Results

### Main outcomes

The results from the cost-effectiveness analyses are shown in Table 2. For the main within ADAPT-Sepsis study analysis (Analysis 1), the daily PCT-guided protocol is dominated (that is, PCT costs more and produces less QALYs than standard care). When only the costs of PCT testing and the difference in number of days of antibiotic treatment are considered using the ADAPT-Sepsis data (Analysis 2), the daily PCT-guided protocol costs £92 (€107) more than standard care with no change in QALYs.

**Table 2:** Probabilistic cost-effectiveness results

Analyses	Time horizon	Inc costs of PCT (€)	Inc QALYs of PCT	ICER	Probability of being cost-effective at €23,220 per QALY	Probability of more QALYs in PCT arm
1. ADAPT-Sepsis (all parameters)	28 days	427	−0.001	Dominated^a^	0.307	0.192
2. ADAPT-Sepsis (only antibiotic treatment duration and PCT testing costs)	28 days	107	Zero	Dominated^a^	Zero	Always equal in both arms
3. Meta-analyzed data (all parameters)	Lifetime	330	0.139	€2384	0.722	0.742
4. Meta-analyzed data (only antibiotic treatment duration and PCT testing costs)	30 days	104	Zero	Dominated^a^	Zero	Always equal in both arms

^a^ Dominated denotes that PCT testing costs more money than current practice whilst not providing health benefits

ICER: incremental cost-effectiveness ratio; Inc: incremental; PCT: procalcitonin; QALYs: quality-adjusted life years

When the ADAPT-Sepsis data are meta-analyzed with other evidence, and a lifetime horizon applied, (Analysis 3) the ICER is £2053 (€2384), based on providing 0.139 more QALYs at an increased cost of £284 (€330), indicating that the use of PCT is cost-effective using NICE’s typical threshold of £20,000 (€23,220). When only the costs of PCT testing and number of days of antibiotic treatment are considered (Analysis 4), the daily PCT-guided protocol costs £89 (€104) more than standard care, respectively.

The results are uncertain however, with PCT testing estimated to produce more QALYs in 19% of simulations in Analysis 1, which just uses ADAPT-Sepsis mortality data, and in 74% of simulations in Analysis 3 which uses meta-analyzed data. The cost-effectiveness planes for Analysis 1 and Analysis 3 are shown in Fig. 1. The larger range in incremental QALYs associated with the daily PCT-guided protocol in Analysis 3 is caused by the increased ramifications of sepsis-related mortality over a lifetime horizon than with a 28-day horizon.

The cost-effectiveness acceptability curves for Analysis 1 and Analysis 3 are shown in Fig. 2.

In Analysis 1, the probability of being cost-effective is largely driven by the relative costs of PCT testing and standard care within the 28-day period, with the daily PCT-guided protocol being associated with lower costs in 31% of simulations. For Analysis 3 there is a high probability that PCT testing is cost-effective compared to standard care, being greater than 72% at all willingness to pay thresholds over £20,000 (€23,220) per QALY gained. There is approximately 26% chance of not being cost-effective at a willingness to pay threshold of €100,000 per QALY gained is due to the possibility that the OS outcome favors standard care (see the meta-analysis results in supplementary material Appendix).

### Sensitivity analysis

When it was assumed that only 10 QALYs would be saved when mortality was presented, the ICER for Analysis 3 increased to £2,602 (€3021) which is still comfortably lower than.

NICE’s £20,000 (€23,220) per QALY threshold. [7].

## Discussion

Previous cost-effectiveness analyses of PCT testing used a short-time horizon or data taken from pre- and post-study designs. A review undertaken by Rojas-Garcis et al. [34] which identified seven cost-effectiveness analyses of PCT testing published between 2000 and 2020 concluded that the majority of studies reported PCT testing to be cost-effective from a societal or healthcare perspective. Three subsequently published cost-effectiveness analyses were identified by the authors of this paper [35–37]. Sligl et al. [37] considered the efficacy of PCT testing within a bundled antimicrobial stewardship program, using data from a pre-and post-study design, and concluded that PCT testing was potentially cost-effective but with substantial uncertainty. Garnfeldt et al. [35] focused on costs in the initial year concluding that a small investment per patient may lead to considerable cost-savings, whilst Mosly et al*.* [36] used a one-year time horizon and concluded that PCT guidance was not likely to be cost-effective.

Thus, to our knowledge, the pre-planned health economic analyses presented here are the first to assess the cost-effectiveness of PCT-guided protocols in reducing antibiotic duration for critically ill adults with sepsis over a lifetime horizon based on the available prospective clinical trial evidence. We have also followed standard UK practices in cost-effectiveness analyses [7] to ensure robustness and transparency of the methods for those wishing to explore further. In addition to looking at the ADAPT-Sepsis study alone, we have performed an updated meta-analysis to incorporate the results from ADAPT-Sepsis into the broader literature base to provide estimates of key parameters considering previous studies. This meta-analysis clearly shows a reduction in antibiotic days. The four analyses we have undertaken show uncertainty in the health benefits associated with PCT testing ranging from a loss of 0.001 QALYs to a gain of 0.139 QALYs with additional costs ranging from €104 to €427 per person. The difference in QALYs is due to the assumed mortality impact of PCT testing, where the point estimate was detrimental in ADAPT-Sepsis, but beneficial when data were meta-analyzed, although in both analyses the change was non-significant, and the non-inferiority margin met in ADAPT-Sepsis. Where no difference in mortality or hospital stay was assumed (Analyses 2 and 4), QALYs were identical and costs modestly increased, by no more than €110 per person. This expenditure may be seen as worthwhile and complementary to hospital antibiotic stewardship programs given the unquantified gain of improved antibiotic stewardship in the general population even if the health of sepsis patients were unaffected [38].

This study has a number of limitations. The original analyses were taken from a UK NHS and PSS perspective and following NICE guidance does not include indirect costs such as productivity losses. This may not be appropriate for different jurisdictions. Furthermore, the costs of resources such as the daily cost of an ICU or antibiotics may vary across countries as could the number of days of PCT testing, the costs of PCT testing or discount rates. Additionally, ICU length of stay and bed utilization may also differ across regions and countries. Therefore, these health economic results may not be generalizable outside of the UK NHS setting.

We did not perform a formal systematic, or targeted, literature review to update the meta-analyses of Wirz et al*.* [1] and Kubo et al*.* [16], but we are not aware of any studies that have been erroneously omitted. Further, only large studies would noticeably change the conclusions from the meta-analyses. The meta-analyses combined studies from different jurisdictions where the control arms could differ. Due to data unavailability, the meta-analyses for lengths of stay and antibiotic usage combined synthesized results from Wirz et al*.* [1] with data from ADAPT-Sepsis rather than the individual study data. Finally, the benefits do not include the longer-term consequences of antibiotic stewardship with reduced risks of antibiotic resistance and potential benefits for patients with difficult to treat infections.

## Conclusions

This economic analysis has shown that PCT-guided antibiotic treatment could be cost-effective and could result in a cost-per QALY well below the threshold for cost-effectiveness used in the UK. Where only differences in antibiotic use and PCT testing are assumed, the increased costs are modest (no more than €110 per person) which may be seen as worthwhile to safely improve antibiotic stewardship for critically ill adult patients with sepsis. This estimate does not include any downstream benefits associated with better antibiotic stewardship that could result in both future cost savings and increased patient health.

## Supplementary Information

Additional file 1

## Data Availability

The datasets used and/or analyzed during the current study are available from the corresponding author on reasonable request.

## Abbreviations

CHEERS: Consolidated Health Economic Evaluation Reporting Standards
CI: Confidence interval
CrI: Credible interval
CRP: C-reactive protein
DDD: Defined daily dose
eMIT: Electronic market information tool
ICER: Incremental cost-effectiveness ratio
ICU: Intensive care unit
NHS: National Health Service
NICE: National Institute for Health and Care Excellence
NIHR: National Institute of Health and Care Research
OS: Overall survival
PCT: Procalcitonin
PSS: Personal social services
QALY: Quality-adjusted life year
RCT: Randomized controlled trial
