# Supplementary material for: Cost-effectiveness of procalcitonin-guided antibiotic duration for hospitalized patients with sepsis
Source: Crit Care. 2025 Nov 28;29:508. doi: 10.1186/s13054-025-05732-w (PMC12661845; doi:10.1186/s13054-025-05732-w)
Supplement: Supplementary file 1 — Additional file 1 [file 13054_2025_5732_MOESM1_ESM.docx]

**Appendix Table 1: The number of times administered within ADAPT-Sepsis, the daily defined dose per administration, and the assumed cost per daily defined dose for antibiotics**

| Drug name | NOTA | Average DDD per use | Assumed cost per DDD (£) | Drug name | NOTA | Average DDD per use | Assumed cost per DDD (£) | Drug name | NOTA | Average DDD per use | Assumed cost per DDD (£) |
| --- | --- | --- | --- | --- | --- | --- | --- | --- | --- | --- | --- |
| Amikacin | 41 | 1.02 | 8.76 | Clarithromycin | 486 | 3.78 | 5.15 | Meropenem | 1235 | 4.18 | 5.54 |
| Amoxicillin | 573 | 4.91 | 2.20 | Clindamycin | 249 | 5.06 | 2.65 | Metronidazole | 778 | 2.99 | 1.38 |
| Azithromycin | 24 | 5.21 | 8.91 | Co-amoxiclav | 937 | 5.63 | 0.30 | Moxifloxacin | 3 | 2.00 | 8.22 |
| Aztreonam | 65 | 3.39 | 37.64 | Co-trimoxazole | 194 | 8.57 | 22.63 | Nitrofurantoin | 26 | 3.82 | 0.17 |
| Benzylpenicillin sodium | 142 | 6.59 | 10.66 | Daptomycin | 12 | 7.44 | 11.22 | Phenoxymethyl-penicillin | 6 | 4.33 | 0.27 |
| Ceflacor | 1 | 2.00 | 0.63 | Doxycycline | 151 | 6.29 | 0.49 | Piperacillin with tazobactam | 2072 | 3.47 | 9.90 |
| Cefalexin | 25 | 4.13 | 0.27 | Ertapenem | 43 | 3.02 | 16.95 | Pivmecillinam hydrochloride | 7 | 7.33 | 1.62 |
| Cefotaxime | 43 | 6.23 | 2.46 | Erythromycin | 42 | 1.76 | 18.59 | Rifampicin | 13 | 4.71 | 9.16 |
| Ceftazidime | 39 | 5.64 | 2.65 | Fidaxomicin | 15 | 7.00 | 135.00 | Rifaximin | 16 | 13.06 | 5.05 |
| Ceftazidime with avibactam | 14 | 4.92 | 257.10 | Flucloxacillin | 337 | 10.64 | 1.10 | Teicoplanin | 484 | 5.86 | 6.15 |
| Ceftolozane with tazobactam | 9 | 13.83 | 201.09 | Fosfomycin | 5 | 8.00 | 64.01 | Temocillin | 66 | 2.51 | 101.80 |
| Ceftriaxone | 146 | 6.53 | 0.88 | Gentamicin | 445 | 2.26 | 6.97 | Tigecycline | 83 | 2.77 | 16.74 |
| Cefuroxime | 170 | 4.34 | 1.78 | Levofloxacin | 145 | 5.79 | 4.27 | Trimethoprim | 21 | 5.00 | 0.17 |
| Chloramphenicol | 8 | 6.22 | 116.43 | Linezolid | 168 | 3.93 | 14.33 | Vancomycin | 597 | 1.30 | 6.50 |
| Ciprofloxacin | 448 | 4.11 | 1.38 | Lymecycline | 1 | 5.00 | 0.20 |  |  |  |  |

DDD – defined daily dose; NOTA – number of times administered

**Appendix Figure 1: Forest plot of overall survival for studies of PCT, OR and 95% confidence interval (CI) of individual studies reported compared with control. Summary outcome reported as posterior median with the 95% credible interval (CrI).**


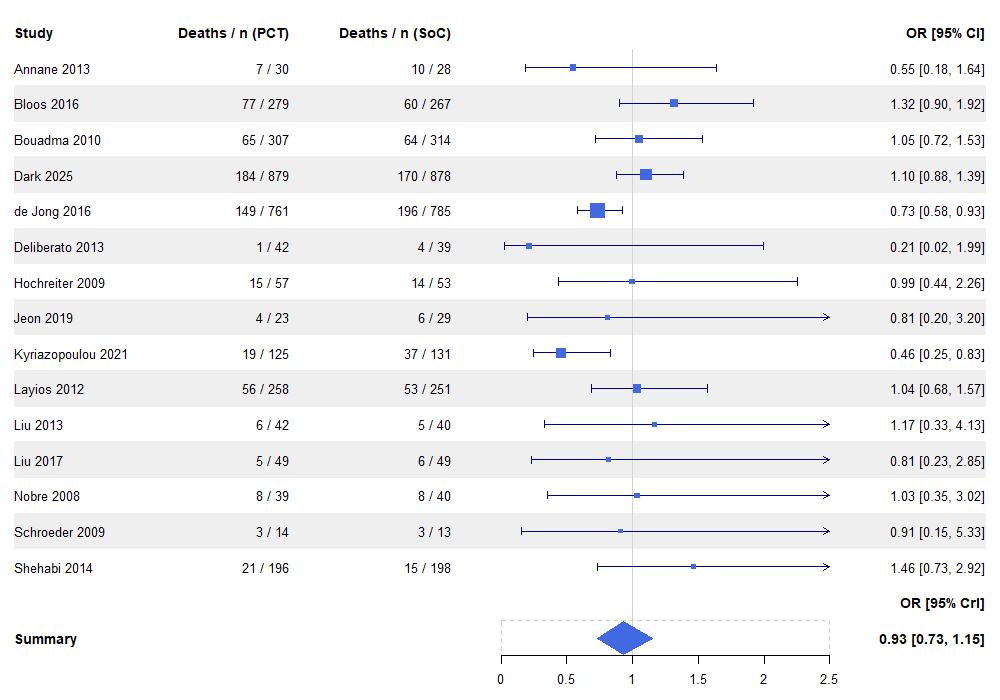


**Appendix Figure 2: Forest plots of change in antibiotic use, change in length of hospital stay, and change in length of ICU stay. Summary outcomes reported as posterior median with the 95% CrI.**

| 1. Change in antibiotic use (days) |
| --- |
| 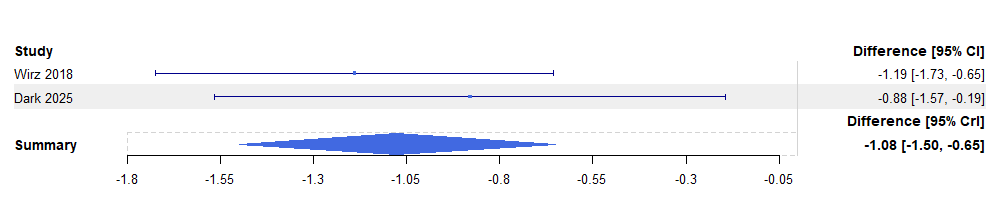 |
| 1. Change in total length of hospital stay (days) |
| 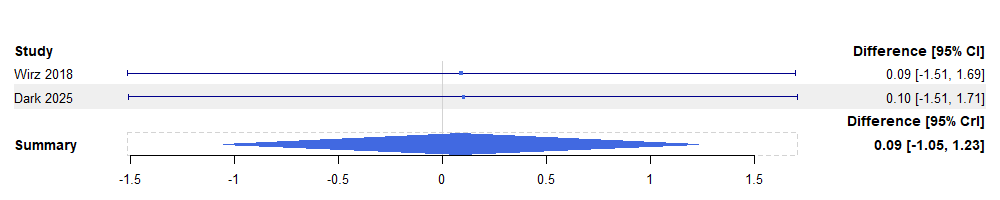 |
| 1. Change in time in intensive care unit (days) |
| 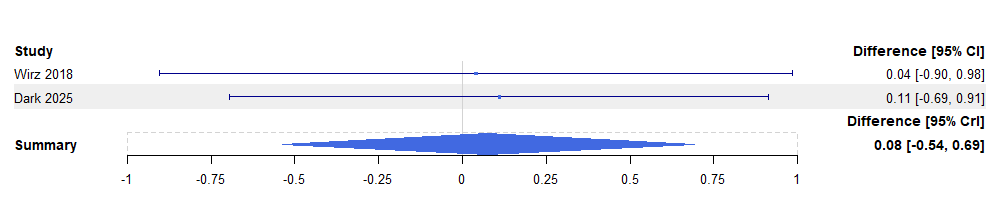 |
